# InterPro in 2022

**DOI:** 10.1093/nar/gkac993

**Published:** 2022-11-09

**Authors:** Typhaine Paysan-Lafosse, Matthias Blum, Sara Chuguransky, Tiago Grego, Beatriz Lázaro Pinto, Gustavo A Salazar, Maxwell L Bileschi, Peer Bork, Alan Bridge, Lucy Colwell, Julian Gough, Daniel H Haft, Ivica Letunić, Aron Marchler-Bauer, Huaiyu Mi, Darren A Natale, Christine A Orengo, Arun P Pandurangan, Catherine Rivoire, Christian J A Sigrist, Ian Sillitoe, Narmada Thanki, Paul D Thomas, Silvio C E Tosatto, Cathy H Wu, Alex Bateman

**Affiliations:** European Molecular Biology Laboratory, European Bioinformatics Institute (EMBL-EBI), Wellcome Genome Campus, Hinxton, Cambridgeshire CB10 1SD, UK; European Molecular Biology Laboratory, European Bioinformatics Institute (EMBL-EBI), Wellcome Genome Campus, Hinxton, Cambridgeshire CB10 1SD, UK; European Molecular Biology Laboratory, European Bioinformatics Institute (EMBL-EBI), Wellcome Genome Campus, Hinxton, Cambridgeshire CB10 1SD, UK; European Molecular Biology Laboratory, European Bioinformatics Institute (EMBL-EBI), Wellcome Genome Campus, Hinxton, Cambridgeshire CB10 1SD, UK; European Molecular Biology Laboratory, European Bioinformatics Institute (EMBL-EBI), Wellcome Genome Campus, Hinxton, Cambridgeshire CB10 1SD, UK; European Molecular Biology Laboratory, European Bioinformatics Institute (EMBL-EBI), Wellcome Genome Campus, Hinxton, Cambridgeshire CB10 1SD, UK; Google Research, Brain team, Cambridge, MA, USA; European Molecular Biology Laboratory, Structural and Computational Biology Unit, Meyerhofstraße 1, 69117 Heidelberg, Germany; Yonsei Frontier Lab (YFL), Yonsei University, 03722 Seoul, South Korea; Department of Bioinformatics, Biocenter, University of Würzburg, 97074 Würzburg, Germany; Swiss-Prot Group, Swiss Institute of Bioinformatics, CMU, 1 rue Michel Servet, CH-1211, Geneva 4, Switzerland; Google Research, Brain team, Cambridge, MA, USA; Department of Chemistry, University of Cambridge, Cambridge, UK; Medical Research Council Laboratory of Molecular Biology, Cambridge Biomedical Campus, Francis Crick Ave, Trumpington, Cambridge CB2 0QH, UK; National Center for Biotechnology Information, National Library of Medicine, National Institutes of Health, 8600 Rockville Pike, Bethesda, MD 20894, USA; Biobyte Solutions GmbH, Bothestr 142, 69126 Heidelberg, Germany; National Center for Biotechnology Information, National Library of Medicine, National Institutes of Health, 8600 Rockville Pike, Bethesda, MD 20894, USA; Division of Bioinformatics, Department of Preventive Medicine, University of Southern California, Los Angeles, CA 90033, USA; Protein Information Resource, Georgetown University Medical Center, Washington, DC 20007, USA; Department of Structural and Molecular Biology, University College London, Gower St, Bloomsbury, London WC1E 6BT, UK; Medical Research Council Laboratory of Molecular Biology, Cambridge Biomedical Campus, Francis Crick Ave, Trumpington, Cambridge CB2 0QH, UK; Department of Biochemistry, Sanger Building, University of Cambridge, Cambridge, UK; Swiss-Prot Group, Swiss Institute of Bioinformatics, CMU, 1 rue Michel Servet, CH-1211, Geneva 4, Switzerland; Swiss-Prot Group, Swiss Institute of Bioinformatics, CMU, 1 rue Michel Servet, CH-1211, Geneva 4, Switzerland; Department of Structural and Molecular Biology, University College London, Gower St, Bloomsbury, London WC1E 6BT, UK; National Center for Biotechnology Information, National Library of Medicine, National Institutes of Health, 8600 Rockville Pike, Bethesda, MD 20894, USA; Division of Bioinformatics, Department of Preventive Medicine, University of Southern California, Los Angeles, CA 90033, USA; Department of Biomedical Sciences, University of Padua, via U. Bassi 58/b, 35131 Padua, Italy; Protein Information Resource, Georgetown University Medical Center, Washington, DC 20007, USA; Center for Bioinformatics and Computational Biology and Protein Information Resource, University of Delaware, Newark, DE 19711, USA; European Molecular Biology Laboratory, European Bioinformatics Institute (EMBL-EBI), Wellcome Genome Campus, Hinxton, Cambridgeshire CB10 1SD, UK

## Abstract

The InterPro database (https://www.ebi.ac.uk/interpro/) provides an integrative classification of protein sequences into families, and identifies functionally important domains and conserved sites. Here, we report recent developments with InterPro (version 90.0) and its associated software, including updates to data content and to the website. These developments extend and enrich the information provided by InterPro, and provide a more user friendly access to the data. Additionally, we have worked on adding Pfam website features to the InterPro website, as the Pfam website will be retired in late 2022. We also show that InterPro's sequence coverage has kept pace with the growth of UniProtKB. Moreover, we report the development of a card game as a method of engaging the non-scientific community. Finally, we discuss the benefits and challenges brought by the use of artificial intelligence for protein structure prediction.

## INTRODUCTION

Advances in genomic technologies together with substantial reductions in the cost of sequencing have enabled the scientific community to generate new sequencing data at an unprecedented scale. To be useful to the scientific community, these hundreds of millions of sequences need to be analysed and characterised, which can often be an issue as the computational time necessary to analyse those sequences is increasing exponentially. To address this challenge, several automated sequence analysis methods have been developed to annotate protein families, domains and functional sites by transferring the information, often from an experimentally characterised sequence, to uncharacterised sequences using predictive diagnostic models (hidden Markov models, patterns, profiles or fingerprints), known as signatures. A number of protein signature databases have been developed, each having their own field of interest (e.g. protein superfamilies, functional and structural domains, orthologous groups).

InterPro combines 13 protein signature databases into one central resource: CATH-Gene3D (1), the Conserved Domains Database (CDD) (2), HAMAP (3), PANTHER (4), Pfam (5), PIRSF (6), PRINTS (7), PROSITE Patterns (8), PROSITE Profiles (8), SMART (9), the Structure–Function Linkage Database (SFLD) (10), SUPERFAMILY (11) and TIGRFAMs (12). Collectively, member databases provide complementary levels of protein annotation, making InterPro the world's most comprehensive resource about protein families, domains, and functional sites. InterPro provides annotations from other resources and tools complementing the member database annotations. These resources include MobiDB-lite (13) for disordered regions, SignalP (14) and Phobius (15) for signal peptide regions, TMHMM (16) for transmembrane regions, coils (17) for coiled-coil regions and AntiFam (18) for spurious proteins.

When signatures from two or more member databases represent the same biological entity, the member database signatures are integrated together into one InterPro entry, reducing redundancy. InterPro entries are annotated with a unique name, short name and InterPro accession number, a descriptive abstract and Gene Ontology (GO) terms (19) that can be consistently assigned to all proteins matched by that entry. An entry type (family, domain, repeat, site or homologous superfamily) is also assigned. Newly created InterPro entries are carefully checked by curators prior to being made available to the public.

## RESULTS

### Content update

#### Member database updates

Like UniProtKB, InterPro follows an 8-week release cycle. Each InterPro release contains new entries, created by integrating member database signatures, and may include one or more member database updates. Since our previous publication that described InterPro 81.0 in 2020 (20), there have been 9 InterPro releases, integrating 10 member database updates: CDD (3.18), CATH-Gene3D (4.3), HAMAP (2020_05, 2021_04), PANTHER (15.0), Pfam (34.0, 35.0), PROSITE Patterns (2021_01, 2022_01) and PROSITE Profiles (2021_01, 2022_01). Over the past 2 years, 1558 member database signatures have been integrated into existing InterPro entries, and 3315 have contributed to the creation of 3,280 new InterPro entries.

InterPro version 90.0 consists of 40 597 entries based on 53 784 integrated member database signatures. As a consequence, the InterPro coverage of sequences in UniProtKB (i.e. the proportion of proteins with one or more InterPro annotations) increased from 81.3% (InterPro version 81.0) to 82.0% (InterPro version 90.0, see Table 1). Although a 0.8% increase may seem small, we should consider that UniProtKB considerably grew in the same period (from ∼189 million sequences to ∼227 million). Therefore, the small increase in InterPro's coverage represents ∼32 million additional sequences with at least one InterPro annotation. We previously reported that 80.3% of sequences in the UniProt Archive (UniParc) were annotated by InterPro (20). During the last 2 years, this coverage slightly decreased to 79.9%.

**Table 1.** Coverage of UniProtKB and UniParc (non-redundant archive of protein sequences) by InterPro entries (version 90.0)

Protein sequence database	Number of sequence entries	Number of sequences entries with one or more matches to InterPro
UniProtKB/reviewed	568 002	549 236 (96.7%)
UniProtKB/unreviewed	226 771 949	185 887 710 (82.0%)
UniProtKB (total)	227 339 951	186 436 946 (82.0%)
Uniparc	517 375 807	413 193 274 (79.9%)

InterPro regularly incorporates member database updates, which allows us to update InterPro entries and provides new signatures for integration. However, updating member databases remains a challenge, especially when it involves substantial data changes, and the overall integration figures often hide a lot of curation work. The percentage of member database signatures integrated into InterPro for each member database is shown in Table 2.

**Table 2.** Release version and number of member database signatures integrated into InterPro version 90.0

Member database	Release number	Total signatures	Integrated signatures
CATH-Gene3D	4.3.0	6631	2712 (40.9%)
CDD	3.18	16212	3817 (23.5%)
HAMAP	2021_04	2383	2379 (99.8%)
PANTHER	15.0	139 691	10 584 (7.6%)
Pfam	35.0	19 632	19 070 (97.1%)
PIRSF	3.10	3285	3236 (98.5%)
PRINTS	42	2106	1944 (92.3%)
PROSITE patterns	2022_01	1311	1283 (97.9%)
PROSITE profiles	2022_01	1326	1258 (94.2%)
SFLD	4	303	158 (52.1%)
SMART	7.1	1312	1267 (96.6%)
SUPERFAMILY	1.75	2019	1642 (81.3%)
TIGRFAMs	15	4488	4434 (98.8%)

PANTHER is a resource for the evolutionary and functional classification of protein-coding genes from all domains of life. InterPro release 91.0 will include an update of the PANTHER database from version 15.0 to 17.0. Since PANTHER release 15.0, PANTHER has provided a second, even more precise method for classifying sequences than the subfamily HMMs: placement in the phylogenetic family tree using the TreeGrafter tool (21). This new implementation has been shown to be more accurate and is five times faster to process than the older, subfamily HMM scoring method.

Historically, both PANTHER family and subfamily HMMs have been integrated in InterPro entries, but the update of the PANTHER subfamilies has always been a challenge for the InterPro curators as it always brings a lot of changes in the signatures. To improve the stability and to make the updates more efficient, we have decided that going forward only PANTHER families’ signatures will be integrated into InterPro entries. However, PANTHER subfamily annotations derived from the tree graft location will still be shown in the list of matches in the protein sequence viewer and the full list of subfamilies will be accessible through the PANTHER family pages.

In 2018 TIGRFAMs relocated to the National Center for Biotechnology Information (NCBI), where it continues to be updated as a component of a larger collection now called NCBIFAMs (12). NCBIFAMs is currently in release 10 (https://ftp.ncbi.nlm.nih.gov/hmm/10.0/). NCBIFAMs includes over 2300 additional models, not yet added to InterPro. These include over 600 models built for accurate identification of bacterial proteins conferring resistance to antibiotics and other antimicrobial agents (22). For the sake of continuity, the collection of HMMs from NCBI appearing in future releases of InterPro will be renamed to ‘*NCBIFAMs (includes TIGRFAMs)*’.

#### Addition of AntiFam

AntiFam contains 250 profile-HMMs that match to common gene mis-predictions that can contaminate sequence databases (18). We have integrated AntiFam version 7.0 in InterProScan 5.55–88.0 and the annotation is shown in the *Otherfeatures* track of InterPro website protein sequence viewer displayed in protein pages.

#### Update of old InterPro entries

For each InterPro release cycle there are two major components. Firstly, the protein update where new data and annotations from UniProt are used to identify InterPro entries that need updating. For example, we can capture a new function for previously uncharacterised protein families by looking at changes to Swiss-Prot description lines. Secondly, one or more member database updates are made to bring them up to their latest version, which affects several entries. These entries are verified by curators, ensuring that the information provided is still accurate and up to date. However, some entries are never affected by those updates and hence can avoid being updated for many years. This can mean that much more is known about the family now than when the function description was written.

In late 2021, we reviewed 626 InterPro entries for which the entry name and description had not been updated and no member database signatures added since 2011, and updated 198 of them (including the update of 54 Pfam entries). Since the beginning of 2022, we have been focusing on InterPro entries with unknown function and we are reviewing a list of entries with known PDB structures and the scientific literature associated, which has been obtained using the PDB/InterPro mapping. So far, 164 InterPro entries have been reviewed out of which 123 InterPro entries (including 86 scientifically characterised proteins) and 69 Pfam entries have been updated.

In the future, we are planning to look at not-recently updated InterPro entries with a short abstract and the absence of reference to the scientific literature, and mapping them to PDB structure papers to obtain more up to date information for the entries.

### InterPro website

The InterPro website (https://www.ebi.ac.uk/interpro/) allows querying and filtering of InterPro data through a feature-rich set of web components developed with the open source React/Redux framework. Through the website, users have the possibility to search by text, protein sequence, domain architecture or to browse through a dataset by applying different filters. New features are continuously added to the website and existing features are enhanced following users’ feedback. In this section we focus on the latest developments made, including the redesign of the website menu, the addition of predicted structural models from RoseTTAFold and AlphaFold, and the integration of the Pfam website features.

#### Home page changes

In the home page, two tabs have been added next to the *Latest entries* tab: *Favourite entries* and *Recent search*. Users can save their favourites entries by clicking on the star symbol next to the InterPro entry name in an InterPro entry page. The list of pinned entries is accessible from the home page in the *Favourite entries* tab. When a new release is made available, the user will receive a notification if one of their favourite entries has changed. When performing a Text search, the text is stored locally and accessible through the *Recent search* tab, allowing the user to retrieve the data results of previous searches.

#### Redesign of webpages menu

**Figure 1.**
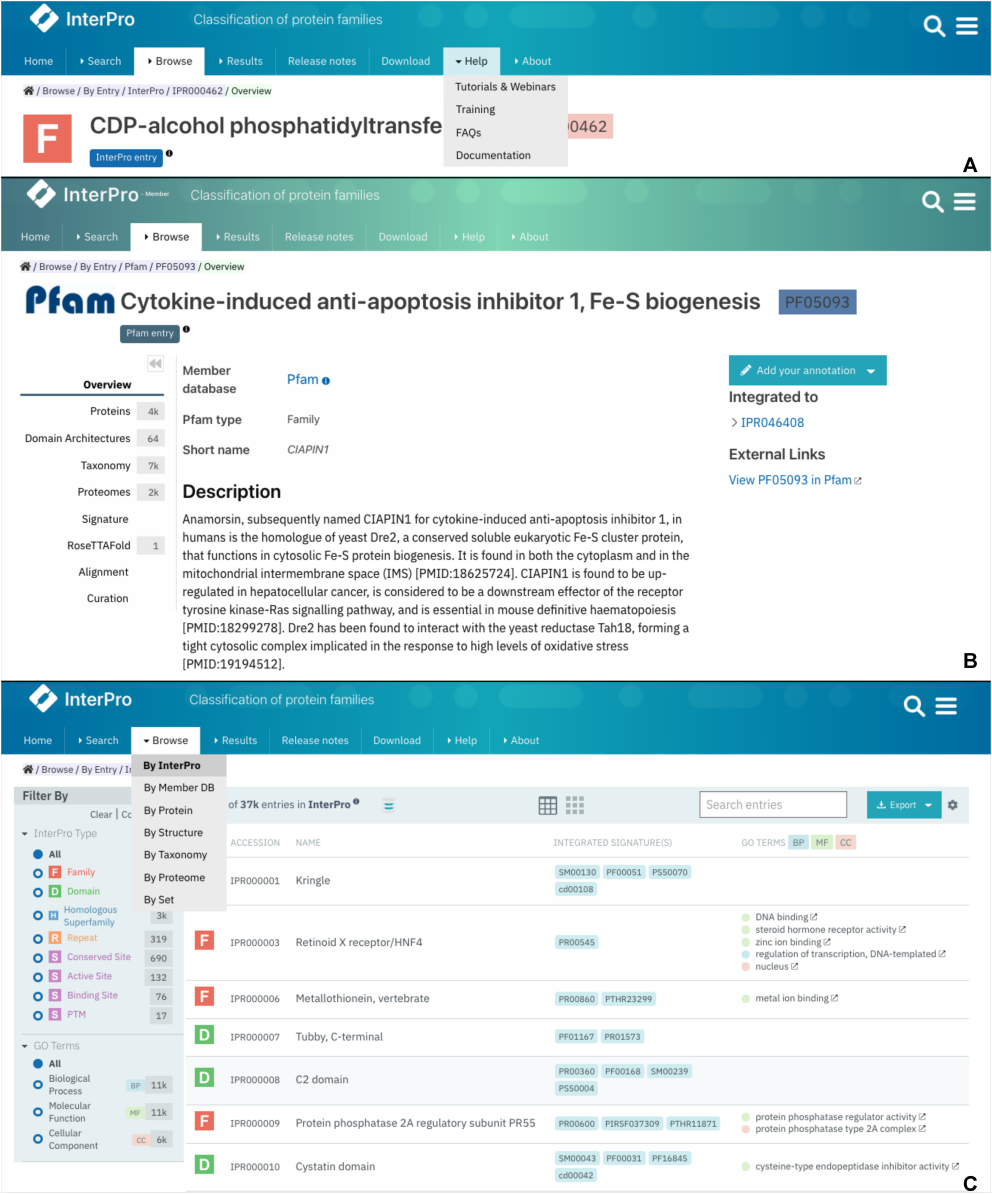
Navigation menus have been updated in multiple places on the InterPro website. The website main menu tabs now expand as dropdowns and breadcrumbs allow to know where pages are located on the website (**A**), the menu in entry pages is displayed on the left-hand side and can be collapsed (**B**), the main data type in the browse feature can be chosen from the *Browse* dropdown menu and filters are now displayed on the left-hand side (**C**).

The website menu, entries menu and browse feature filtering options have been redesigned for easier access to the data. Dropdown menus have been added to the website main menu tabs, as illustrated in Figure 1A. They allow easy access to subsections and replace the tabs previously displayed at the top of the relevant pages. We have also added a breadcrumbs component at the top of each page, so it is easy to check where on the website the current page is located (Figure 1A).

Previously, when the number of tabs available in an entry page could not fit in a single line, the menu was expanded to two lines, making it confusing. To resolve this issue, we have redesigned the menu, it is now displayed as a side menu on the left-hand side of entities. It can be expanded or collapsed using the double arrows symbol located at the top of the menu for an easier visualisation of the page content, as shown in Figure 1B.

The browse feature has also been redesigned following user testing feedback. The main endpoint tabs (Entry, Protein, Structure, Taxonomy, Proteome, Set) have been moved to a dropdown list displayed when hovering over the *Browse* tab in the website navigation menu. The Entry endpoint has been split into two entities: InterPro entries (*Browse by InterPro*) and member database signatures (*Browse by Member DB*), as shown in Figure 1C.

#### Structural model predictions

The field of protein structure prediction has greatly advanced over recent years such that deep-learning based methods are now able to predict high quality de novo protein structures.

##### RoseTTAFold

Structure models and contact maps have been created for the majority of Pfam families that do not have a structure in the PDB. They are available under the *RoseTTAFold* tab of InterPro entry (e.g. IPR031639) and Pfam signature pages (e.g. PF16915). The models are generated by the Baker laboratory using RoseTTAFold (23).

The 3D structure of the model is displayed in the Mol* Viewer (24) and the residues are coloured using the predicted Local Distance Difference Test (pLDDT) score (25), with a gradient going from blue (high confidence) to red (low confidence). Below the 3D viewer, the heatmap visualisation displays the residue contacts. Hovering on the heatmap highlights the contacts in the 3D structural model. Additionally, the contact map information is displayed for the Pfam family SEED alignment. Hovering or clicking on a contact position highlights its connection to other residues in the alignment as well as on the 3D structure.

##### AlphaFold

AlphaFold 2.0 (26) has revolutionised structure prediction enabling the rapid creation of high-quality models across many model organisms. We expect these models to drive forward the field of molecular biology and biomedical research. DeepMind and EMBL’s European Bioinformatics Institute (EMBL-EBI) have launched the AlphaFold Protein Structure Database (AlphaFold DB) (27), a joint project to openly and freely share millions of AlphaFold protein structure predictions with the scientific community.

We provide two entry points within InterPro to the AlphaFold structural models. Firstly, when looking at a protein page, if a model is available, clicking on the *AlphaFold* tab allows one to view the model's 3D structure. The second entry point is via the *AlphaFold* tab in InterPro entry pages. In this case, the *AlphaFold* tab shows an example AlphaFold model and a table below which displays other models that are available for that entry.

#### Member database signature logos

The representative model that defines a member database signature can be visualised as a logo, using Skylign (28). This is displayed under the *Signature* tab in member database entry pages. This feature was previously only available for the Pfam database. It is now also available for PANTHER, PIRSF, SFLD and TIGRFAMs signatures.

#### Sequence search improvements

Sequence searches against InterPro member databases allow prediction of the function, domains and sites of proteins. This feature is powered by our servers using InterProScan as a web service. New functionalities have been added that allows users to study, save, and update the results of previous searches.

On the sequence search result page, the user can visualise previously submitted searches, by default saved for seven days on our servers. If a user wants to keep the results for a longer time, a results file in JSON format can be downloaded. Alternatively, the InterPro website offers the option to save that file in the browser. Sequence search result files, whether obtained from the web service or generated by a local InterProScan instance, can be uploaded to the website at a later date allowing the user to inspect the results in the protein sequence viewer. This feature can, for example, be used to generate images for scientific publications.

Saved or imported results will eventually become outdated as new versions of InterPro data are released. Therefore, when the website identifies a mismatch between versions, a warning is included in the results and a button is available to re-run the job, using the same sequence and options, but now with the most recent InterPro release.

#### Protein sequence viewer

**Figure 2.**
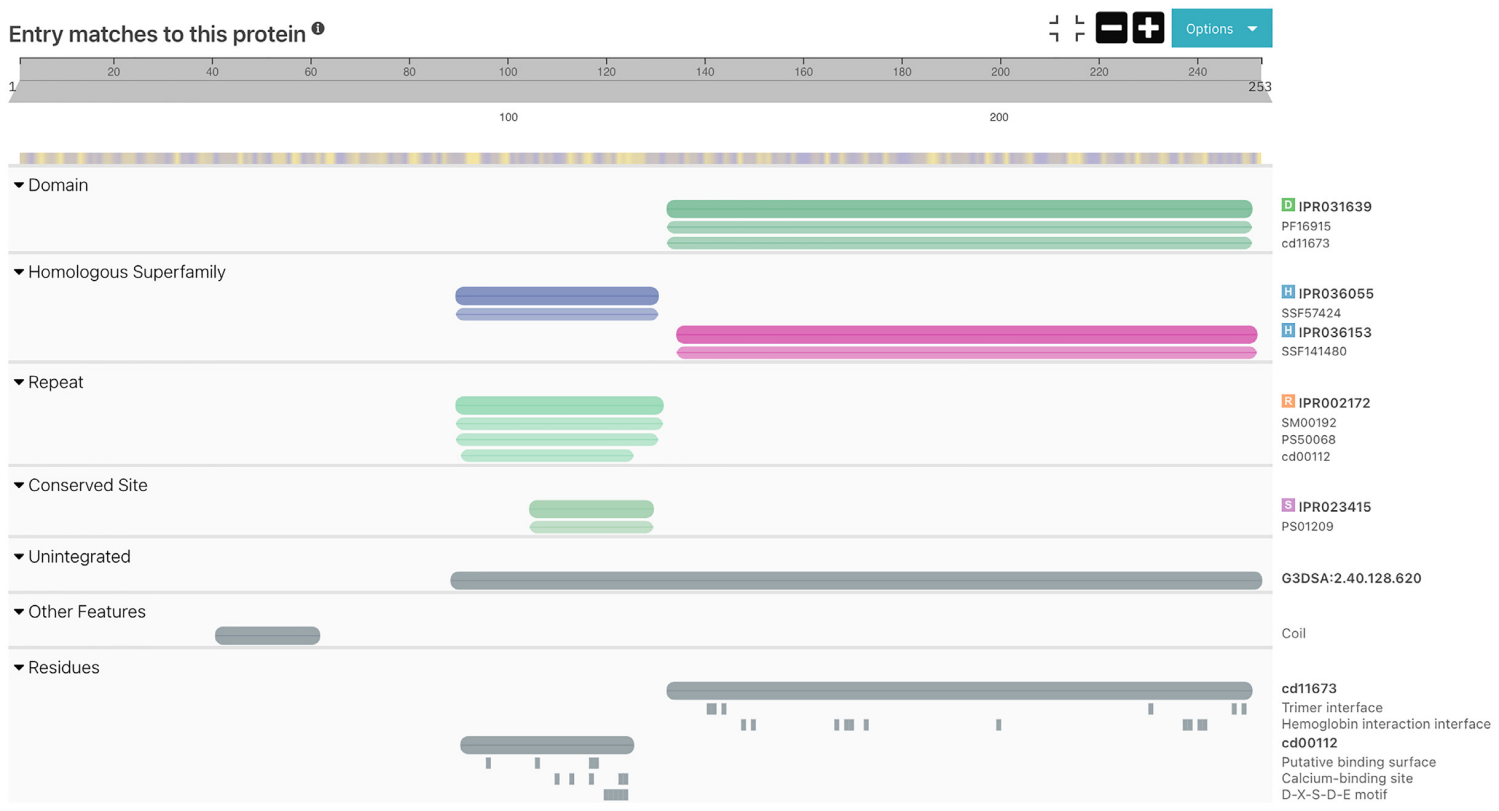
Protein sequence viewer with the *Residues* section for UniProtKB: P18207.

A new *Residues* section has been added to the Protein sequence viewer (see Figure 2). It groups all the residue information, provided by the CDD, SFLD and PIRSR (29) member databases, in one location.

#### Short names display

In the *Options* menu of the sequence viewer in protein pages, we offer the possibility to display entries by *Accession*, *Name* and now *Short name* labels. While accessions are the stable identifiers for InterPro and member databases, names and short names are more descriptive of protein families and often used by biologists when searching for information. We have also added the option to display the Accession, Name or Short name labels in the graph showing the relationship between different methods included in a set (e.g. CDD cl00014).

#### Integration of the Pfam website features

After many years of good and faithful service, it was decided to retire the Pfam website due to its ageing codebase and the lack of resources to maintain it in the long term. Ahead of the decommission, we have made sure that all the key features available in the Pfam website have been implemented in the InterPro website. Below we present key functionality that has been added to the InterPro website: the taxonomy sunburst representation and improvements to the domain architecture visualisation. Other Pfam features have been added to Pfam entry pages: a *Curation* tab and Wikipedia information. Literature references have also been added to Pfam set pages.

##### Taxonomy sunburst

InterPro entry pages and member database entry pages have a *Taxonomy* subpage. The list of species represented in these entries is based on data from UniProt taxonomy. Previously the *Taxonomy* subpage offered three different views: *All species* table, *Taxonomy tree* and a *Key Species* table. Sunburst is a new visualisation of taxonomy data in InterPro. A sunburst visualisation is a multilayer pie graph that compresses a lot of hierarchical information into a limited space. It shows at a glance the proportions of a variable of interest at different levels of the hierarchy.

**Figure 3.**
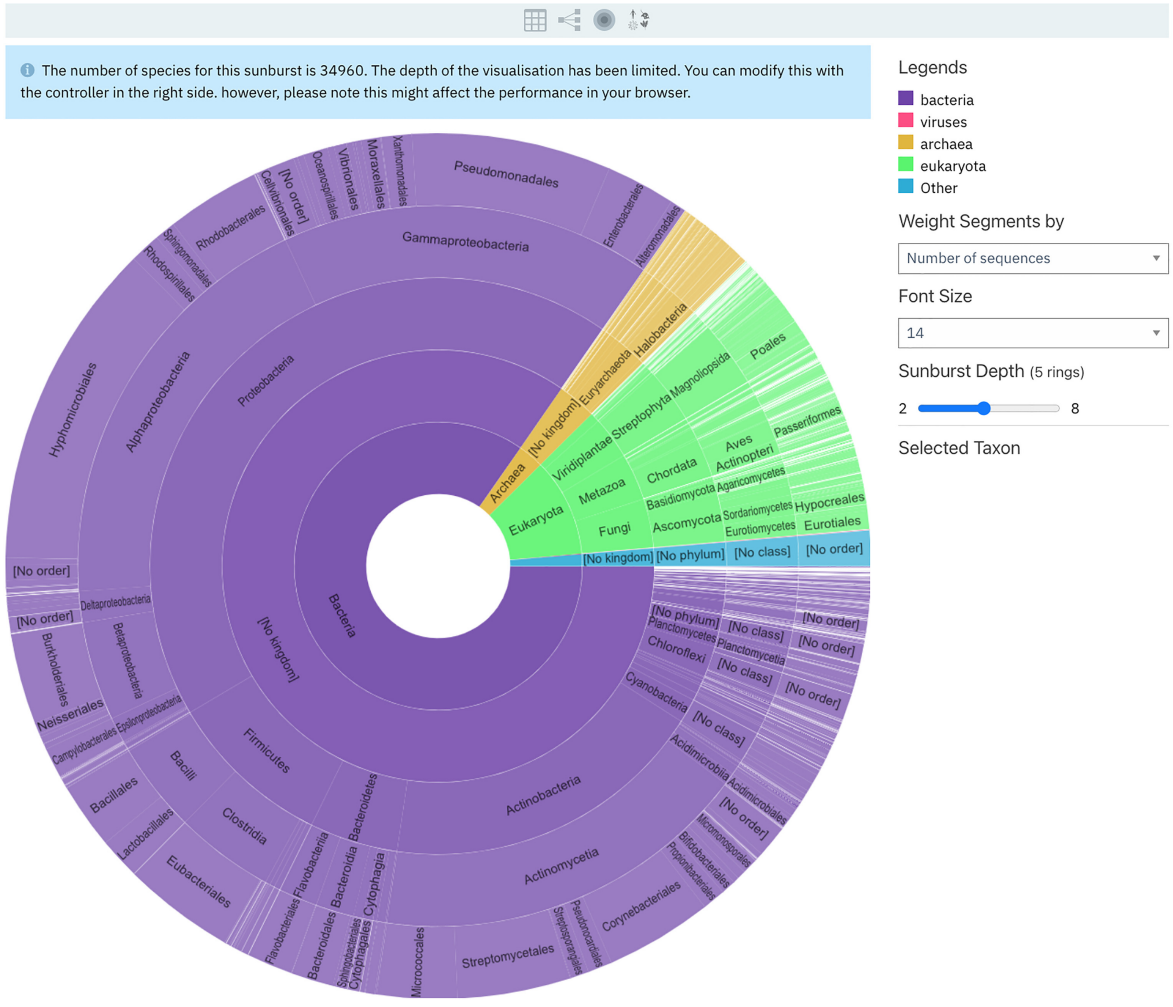
Taxonomy sunburst view for PF00120.

The sunburst in InterPro displays the taxonomy distribution of the proteins matching the entry, from the least specific at the centre to more specific going towards the outside. For example, in Figure 3, the user can infer from the graph that for Pfam PF00120, most of the matches are in bacteria (mainly purple colour), and more specifically in proteobacteria.

A range of options can be selected to customise the view: the segment size can be adjusted based on the number of sequences matching a taxon (default) or by the number of species per taxon, and the sunburst depth can be adjusted between 2 and 8 rings.

##### Redesign of the domain architectures feature

Domain architectures provide information about the different domain arrangements for the proteins matched by an entry based on Pfam signatures. This information can be found under the *Domain architecture* tab of an InterPro entry or member database entry, the *Similar proteins* tab of a protein, and the *r*esults of a *Search by Domain architecture*.

**Figure 4.**
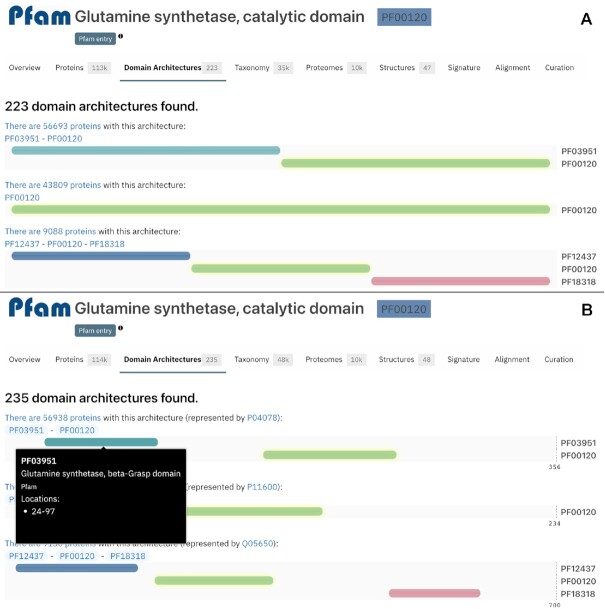
Domain architectures display changes between InterPro 87.0 (**A**) and InterPro 88.0 (**B**) for PF00120.

Previously, the domains throughout the protein length were displayed next to each other with equal sizes, as shown in Figure 4A. To display a more biologically correct visualisation, the domain size is now displayed based on the real length of the domain, using a protein of reference. When hovering over a domain, more details are available in a tooltip, including the domain's position, as shown in Figure 4B.

### API

The InterPro API allows programmatic access to InterPro data, offering scientists the possibility to run further bioinformatics analysis to suit their research needs. It is accessible via the following link: https://www.ebi.ac.uk/interpro/api/.

Throughout the last 2 years the API has been regularly updated to provide the data required for all the functionalities mentioned above. Most updates involve minor changes to the underlying databases to, for example, include new data, or optimise its access and avoid deterioration of the API performance.

We have also carried out periodic maintenance of its codebase, making sure that necessary dependency updates are completed to minimise the security risk of our infrastructure.

#### API documentation update

The InterPro API documentation consists of general documentation available on GitHub (https://github.com/ProteinsWebTeam/interpro7-api/tree/master/docs) and Swagger API documentation (https://www.ebi.ac.uk/interpro/api/static_files/swagger/) allowing the application of a range of modifiers to the different API data types to filter the output data. In the last two years we have updated the documentation, including the addition of examples of modifiers that can be used to filter the data.

### Outreach and communication

In the last two years, InterPro has been active in engaging with scientific and non-scientific audiences via Twitter, blogging, and game development.

The InterPro Twitter feed (@InterproDB), first introduced in 2012, was initially used only to announce new InterPro releases. Since September 2020, InterPro has increased its social media presence by tweeting about new features, job opportunities, and protein focus articles written by members of the InterPro team. This engagement has led to the increase of the number of followers from 1014 in July 2020 to 1996 in July 2022.

Additionally, since InterPro 83.0 (October 2020), we have introduced the *Release* blog posts. For each release, they highlight new developments or updates that have been made to the InterPro website and API developments.

**Figure 5.**
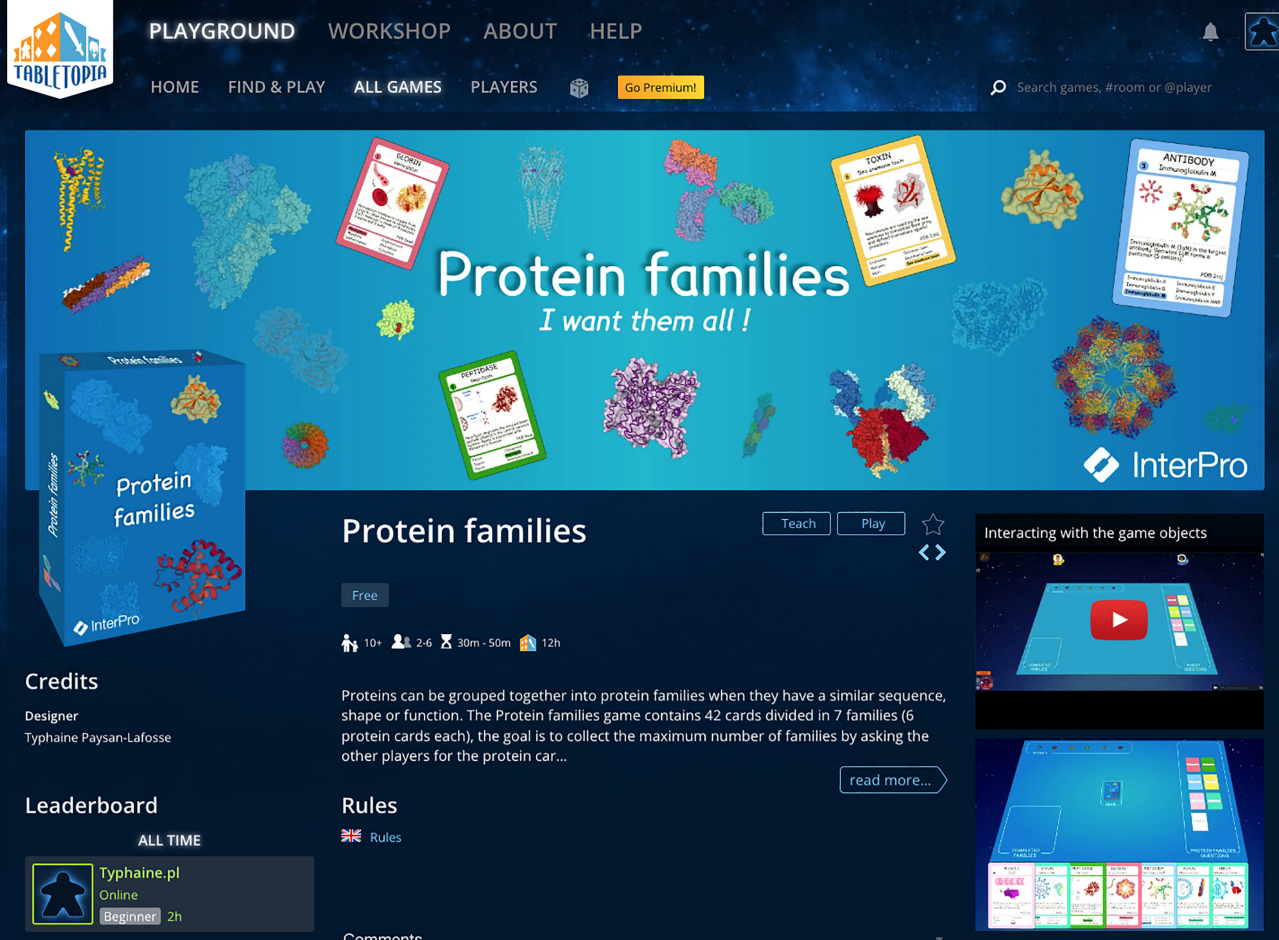
Protein family game landing page in Tabletopia.

Furthermore, we have developed a public engagement activity in the form of a card game: Protein families. The main objective of this game is for the players to have fun whilst learning new things about proteins, without necessarily being aware of it. The game contains 42 cards divided in 7 families (6 protein cards each), the goal is to collect the maximum number of families by asking the other players for the protein cards you are missing in your hand to complete your families. Through this game players can discover that proteins are related and can be classified in families depending on their functionality and/or 3D structure. They are also learning interesting information about the proteins, and explore the beauty of protein structures through the 3D visualisation. The game has been developed through an iterative process asking for feedback from scientists and non-scientists audiences through surveys and play testing. The Protein families game is available online in the Tabletopia game platform (https://tabletopia.com/games/protein-families), as illustrated in Figure 5, and as a physical card game. The Protein families card game is part of EMBL-EBI’s public engagement in STEM (science, technology, engineering, and maths), subjects related to the activities of the institute. This scheme aims to bring together EMBL-EBI’s staff and students, working and studying in the STEM sector, with the lay public.

## DISCUSSION

Over the last two years we have carried out extensive development of InterPro. On the curation side, despite the continous growth of UniProtKB, we have continued to review and integrate signatures, leading to a slight increase in the coverage of UniProtKB. On the web development side, we have redesigned several of the InterPro website features and developed new functionalities previously found in the Pfam website. InterPro now provides the sole archival source and website for both the PRINTS and SFLD databases. InterPro will also provide website access to the Pfam database in the future. Given the limited options for funding biological data resources we see a growing role for InterPro to provide an important archival function as well as a centralised web interface for protein domain and family resources.

Artificial intelligence (AI) and deep learning (DL) methods are becoming increasingly popular and more and more accurate for a wide variety of tasks. Applying AI-based methods for the prediction of protein structures, such as AlphaFold2 and RoseTTAFold, has led to an impressive step forward in the field of molecular biology and could allow the prediction of protein-protein interactions, opening many new opportunities for disease treatment and drug discovery (30). Additionally, DL methods can also be used to predict protein functions. A Google Research team is working on the development of ProtENN, a deep-learning method predicting functional annotations for unaligned amino acid sequences using Pfam families as a training set (31). With Deep Learning approaches beginning to out-perform existing alignment-based approaches like profile-HMMs we can envision a shift in the tools that are used for protein domain and family classification in the coming years. Protein function prediction using DL methods opens the door to a new era of protein classification, but at the same time brings challenges for the integration of these new models in a resource like InterPro, as we will need to ensure the model's accuracy to make sure we do not lose quality or quantity of annotations. Retraining DL models frequently could lead to improvements but also volatility in results. We are excited for what the future holds and are quietly hopeful that researchers in protein classification will have their own AlphaFold moment in the coming years.

## DATA AVAILABILITY

All data is freely available for browsing and download via the InterPro website https://www.ebi.ac.uk/interpro/.
